# Two CIDP Variants Patients With Anti-Caspr1 Antibodies in South China

**DOI:** 10.3389/fimmu.2022.844036

**Published:** 2022-03-11

**Authors:** Chong Li, Hui Zheng, Chao Yuan, Yanran Li, Yafang Hu, Haishan Jiang

**Affiliations:** Department of Neurology, Nanfang Hospital, Southern Medical University, Guangzhou, China

**Keywords:** polyradiculoneuropathy, chronic inflammatory demyelinating, cell adhesion molecules, neuronal, contactins

## Abstract

**Background and Objectives:**

Chronic inflammatory demyelinating polyradiculoneuropathy (CIDP) is considered an immune-mediated heterogeneous disease that involves both cellular and humoral immunity. The advent of the new concept of node-paranodopathy in recent years has boosted the identification of more antibody-positive CIDP variants patients. Cases of Caspr1 autoantibodies are the least common. Here, we reported two patients with Caspr1 autoantibodies and summarized their clinical features and treatment responses.

**Methods:**

Do statistical analyses on the clinical manifestations and laboratory examinations obtained from two patients identified in this study, and eight patients with anti-Caspr1 antibodies reported in previous research. And based on the developed scoring standard, draw the radar charts and line graphs.

**Results:**

Similar to other studies, the two patients we mentioned had a subacute and severe onset, distal phenotype, sensory ataxia, and severe pain. Differently, they had severe pain accompanying cold sense and coarse tremor in both hands, which may be a typical symptom for the anti-Caspr1 positive patient in south China. And we drew the line and radar graph for two China patients based on five aspects, muscle strength, sensory nerve, cranial nerve, laboratory tests, and NCS examinations. The two visual data charts offered new complementary means for the diagnostic assessment of CIDP variants.

**Conclusion:**

Pain with cold sense, coarse tremor in hands, and CSF protein levels greater than 3g/L may be the source of the distinct symptoms observed in patients with anti-Caspr1 autoantibodies in south China.

## Introduction

Nodes of Ranvier are classified into nodal, paranodal, and juxtaparanodal compartments, each with its unique set of adhesion proteins and ion channels.Neurofascin-155 (NF155), contactin-1 (CNTN1), and contactin-associated protein 1 (CASPR1) form a link between myelin sheath and axon at the paranodes (1). Some studies in recent years have shown that auto-antibodies are targeted at the proteins of paranodes in a limited percentage of CIDP patients (2–4). These antibodies can disrupt axon-glial interactions without causing macrophage-induced demyelination, which is a characteristic of conventional CIDP (5, 6). The clinical manifestations and therapeutic responses of antibody-positive patients are significantly different from those of antibody-negative patients. The antibody-positive patients have a subacute or acute onset, a distal phenotype, sensory ataxia and tremor, and inadequate response to intravenous immunoglobulins (IVIG).

The first patients of Caspr1 autoantibodies were identified in 2015 (2), yet only 8 cases have been reported so far. This is in line with previous reports that the prevalence of anti-Caspr1 antibodies was low, about 2% (3) and 0.2% (4). Accordingly, the clinical characteristics of patients with positive anti-CARSP1 antibodies have not been adequately established. The two patients in the first study had severe neuropathic pain, but there was none reported in the other 6 patients. Two patients with anti-Caspr1 antibodies seemed to have a higher incidence of cranial nerve involvement and respiratory failure (4), whereas none of the other 6 patients had similar symptoms.

This study identified two patients with anti-Caspr1 antibodies, stressing some specific symptoms and therapies, such as neuropathic pain with cold sensation, coarse tremor in the hands, and nerve conduction study (NCS) examinations. Furthermore, we summarized clinical features and treatment responses of Caspr1 autoantibodies to better understand the disease and manage patients.

## Method

### Patients

We found two patients had similar symptoms like the reported patients by the reported patient by Doppler et al. (2), and we examined the antibodies and confirmed the Caspr1 positive(see in Supplementary files), indicating Caspr1-positive CIDP could have common symptoms. Based on EFNS/PNS criteria (7), both patients were diagnosed with CIDP variants. So we decided to summarize the features of these patients, performing statistical analyses on the clinical manifestations and laboratory examinations obtained from two patients identified in this study, and eight patients with anti-Caspr1 antibodies reported in previous research (2–4, 8). This study was approved by the Ethics Committee of Nanfang Hospital, Southern Medical University.

### Cell-based assay (CBA) assay

Human embryonic kidney cells were plated onto poly-L-lysine coated glass coverslips in 24-well plates at a density of 50 000 cells/wells and were transiently transfected with Caspr1 constructs (NM.003632.3) using pcDNA3.1-c-eGFP. The day after, cells were incubated for 24 h. Then cells were fixed, permeabilized, and incubated for 2 h with serum diluted at 1:10 in PBS at 37°C. After several washes with PBS, cells were incubated with goat antibodies against human IgG H&L (1:200; DyLight^®^ 550) (ab96908) for 1 h at 37°C.

### Scoring Criterion, Radar Chart, and Line Graphs

**Figure 1:**
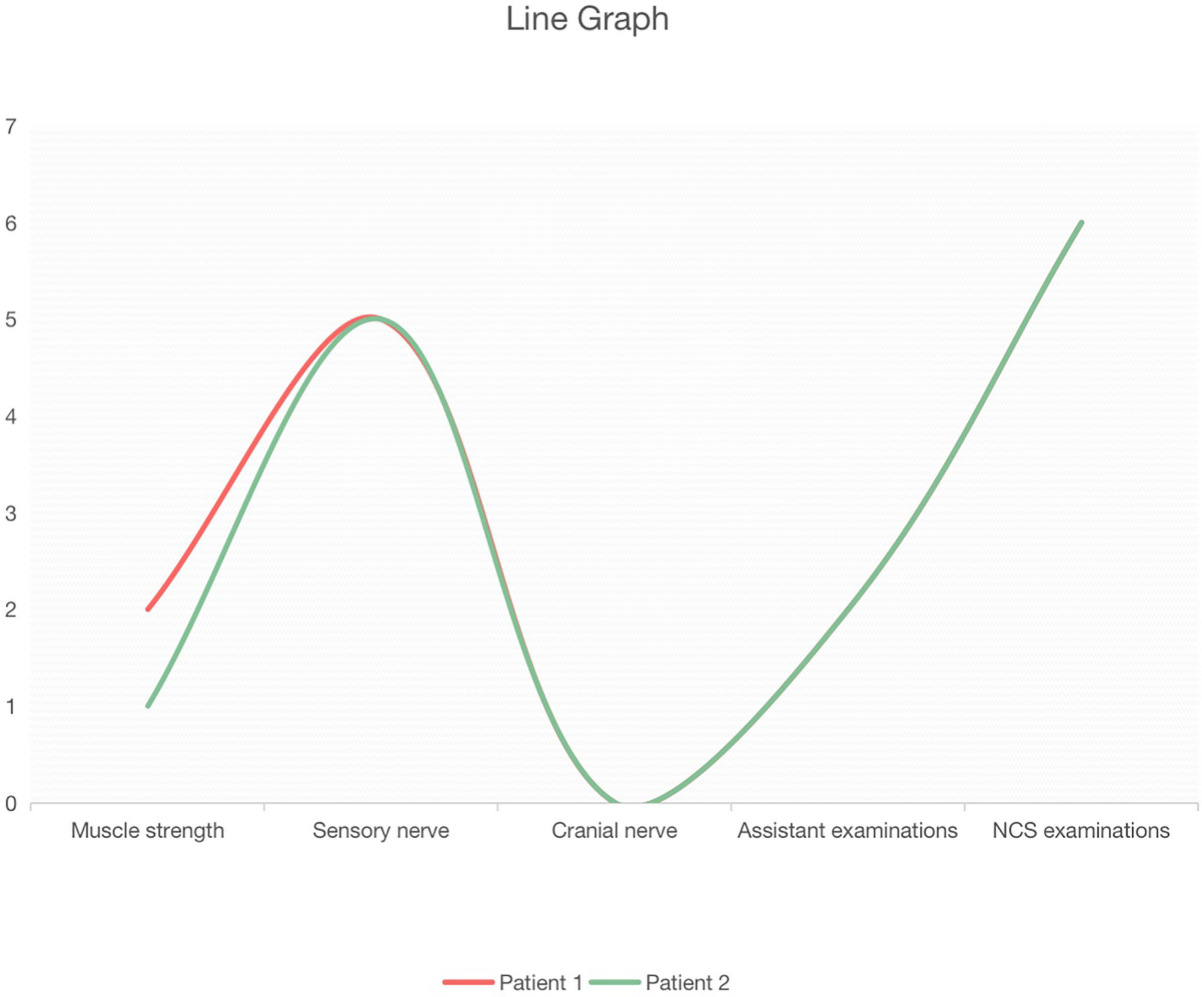
Line graph of the two patients based on the scoring standard.

We developed a scoring standard with 5 sections: muscle strength, sensory nerve, cranial nerve, laboratory tests, and nerve conduction study (NCS) tests. The contents of each section are shown in **Figure 1**. The muscle strength section included muscle strength of the proximal upper limb, distal upper limb, proximal lower limb, distal lower limb, and torso muscle. The sensory nerve section included neuropathic pain, cold sense, sensory deficiency, sensory ataxia, and tremor. The cranial nerve section included respiratory failure and other nerve involvement. The laboratory tests included cerebrospinal fluid the level of CSF protein and anti-Caspr1 titer. Finally, the NCS examinations section included prolonged distal motor latency (DML), reduced conduction velocity (CV), decreased compound muscle action potential (CMAP) or sensory nerve action potential (SNAP), prolonged F wave, conduction blocks, temporal dispersion, and increased CV, after steroids therapy in NCS examinations.

The evaluation of muscle strength was based on the Lovett scale for muscle strength, where the patient was assigned +1 score for every one-point decrease in any muscle. In the sensory nerve and the cranial nerve sections, the patients were assigned a +1 score if they had any symptoms. In the laboratory tests, the patients scored a point if their CSF protein level was higher than 3g/L or if they had an anti-Caspr1 antibody. The patients were assigned a point if their CNS examination was positive. Then, the Radar Maps and Line Charts for both two patients were produced.

### Statistical Analysis

Clinical data were presented as a percentage (%) or as a mean (min-max). Categorical variables were not compared using the Chi-square test or Fisher exact test because the samples were too small.

## Results

### Clinical Features of the Two Patients

Patient 1 was a 44-year-old male who developed sensory deficits, pareses, and severe pain with a cold sensation in his distal limbs in February 2020 without a precursor infection history. In addition, the patient had sensory ataxia and coarse tremor in the hands. Symptoms were rapidly progressive, and the patient had difficulty walking within a few weeks. There were no autonomic symptoms and cranial nerve involvement. His CSF protein level was high at 3.3g/L. At the onset of the disease, nerve conduction tests revealed features of a demyelinating neuropathy, such as decreased nerve conduction velocity, prolonged distal motor latency, F waves and reduced compound muscle, and sensory nerve action potentials. Symptoms were continuously progressive until the patient was largely confined to a wheelchair and could only take a few steps with crutches. high dose glucocorticoid therapy and sequential glucocorticoid treatment were both effective, and the patient showed marked improvement. Pareses and sensory deficits ameliorated, and the patient was eventually able to walk without aid. The detection of CNTN1, CNTN2, Caspr2, NF155, NF186, and antiganglioside antibodies had been also tested in Nanfang Hospital’s Neurology Laboratory and these were negative.

Patient 2 was a 59-year-old female who developed pain with a cold sensation and numbness in the distal limbs, followed by pareses in August 2019, after a respiratory infection. Pareses and sensory deficits worsened, and the patient developed a major lower limb disability and needed aid to walk. She also had sensory ataxia and manifested coarse tremors in the hands. She presented with no autonomic symptoms and cranial nerve involvement. Her CSF test revealed a high protein level at 3.2g/L. A magnetic resonance imaging (MRI) scan of the lumbosacral and brachial plexus indicated that the spinal roots were enlarged on both sides. Nerve conduction tests showed prolonged distal motor latency, decreased nerve conduction velocities, and amplitude prolonged F waves, reduced compound muscle, and sensory nerve action potentials were also be detected. Symptoms were continuously progressive. The patient was refractory to IVIG. The medical conditions ameliorated after treatment with high-dose glucocorticoid therapy and glucocorticoid sequential therapy. The detection of CNTN1, CNTN2, Caspr2, NF155, NF186, and antiganglioside antibodies had been also tested in Nanfang Hospital’s Neurology Laboratory and these were negative.

**Figure 2:**
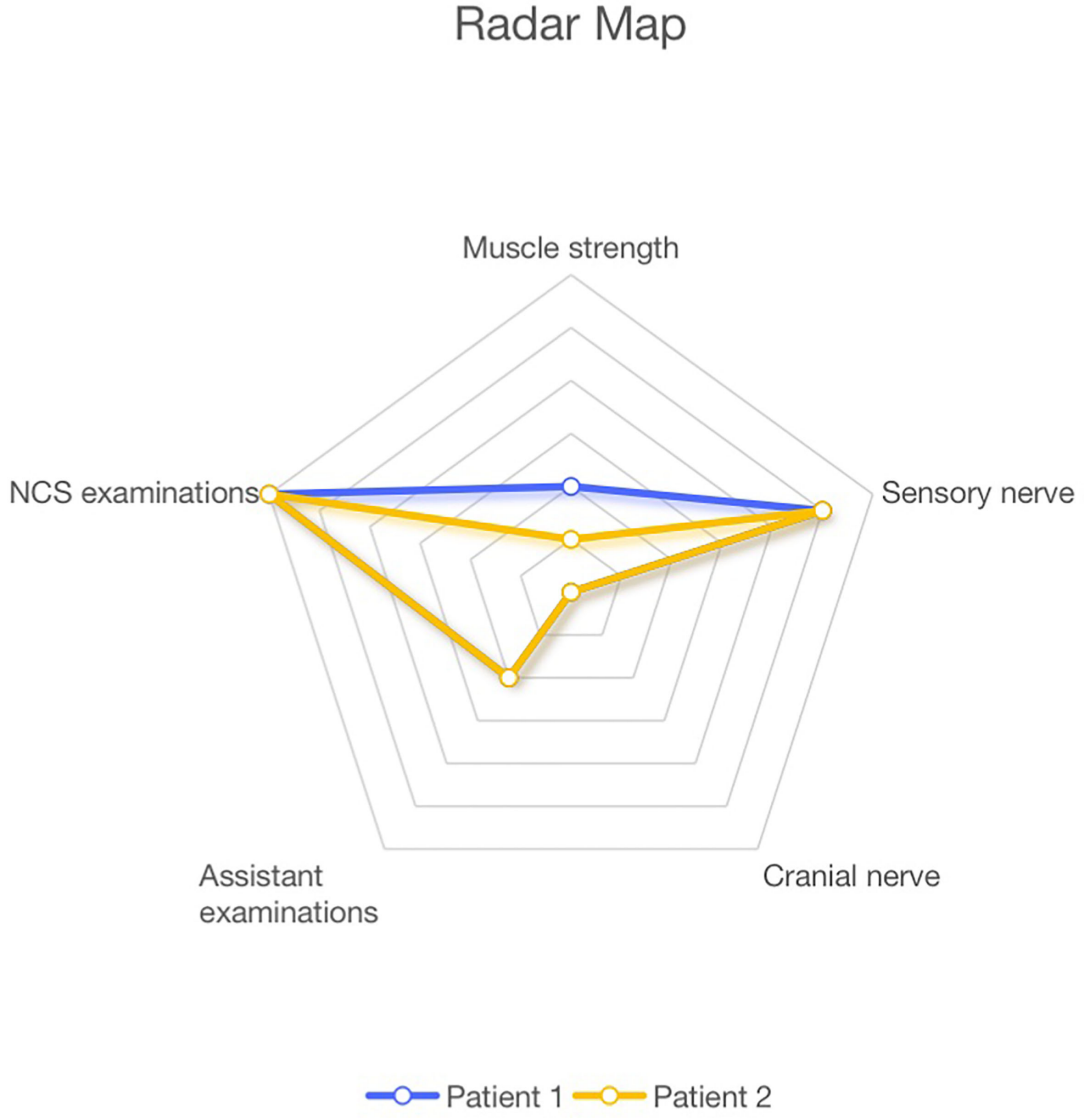
Radar graph of the two patients based on the scoring standard.

The prominent clinical features and the score for each patient are shown in **Table 1**, whereas the radar charts and line graphs for each patient are depicted in **Figures 1** and **2**. We note with emphasis the similarity in scores of each section for the two patients such that the images of radar charts and line graphs are nearly identical (**Figure 1**). Both patients had severe neuropathic pain with cold sensation and coarse tremors in the hands. At the exacerbation stage, the NCS examination revealed that both patients had prolonged DML with an increase of 100% - 200%, reduced conduction amplitude (CA), with a decrease of 80% - 90%, and NCV with a decrease of 60% - 70%, dictating demyelination and axonal degeneration, which were ameliorated after steroids therapy. Notably, the rate of improvement in CV was better than in the other clinical features. It was found that in patients with Caspr1 antibodies, sensory nerve symptoms were more severe than motor nerve symptoms, and NCS examinations yielded several positive results.

**Table 1:** Clinical features and scores of patients.

Sections	Patient 1	Patient 2
		
**Muscle strength**		
** Proximal upper limb**	0	0
**Distal upper limb**	1	0
** Proximal lower limb**	0	0
**Distal lower limb**	1	1
**Torso**	0	0
Score*	2	1
*: +1 score for every one-point decrease in any muscle		
**Sensory nerve**		
** Neuropathic pain**	1	1
** Cold sense**	1	1
** Sensory deficiency**	1	1
** Sensory ataxia**	1	1
** Tremor**	1	1
Score*	5	5
*: +1 score for any symptoms		
**Cranial nerve**		
**Respiratory deficiency**	0	0
**Other nerve involvement**	0	0
Score*	0	0
*: +1 score for any symptoms		
**Laboratory tests**		
** CSF protein**	1	1
** Anti-Caspr1 titer**	1	1
Score*	2	2
*: **+1 score if CSF protein level was higher than 3g/L or if the patient had an anti-Caspr1 antibody**		
**NCS examinations**		
**Prolonged DML**	1	1
**Reduced CV**	1	1
**Reduced CMAP or SNAP**	1	1
**Prolonged F wave**	1	1
**Conduction blocks**	1	1
**Temporal dispersion**	0	0
**Increased CV after steroids therapy**	1	1
Score*	6	6
*: a point if the CNS examination was positive		

CSF, cerebrospinal fluid; CV, conduction velocity; CMAP, compound muscle action potential; SNAP, sensory nerve action potential; DML, distal motor latency; NCS, nerve conduction study.

### Analysis and Comparison of Patients With Anti-Caspr1 IgG4 Antibodies

We used IgG4 assay as an alternative for the isotype test of anti-Caspr1 antibodies which was not performed. Since the antibodies were of the IgG4 isotype, neither patient responded to IVIG treatments. We gathered all eight patients who had been confirmed to have anti-Caspr1 IgG4 antibodies and compared them to the two patients we reported. (**Table 2**).

**Table 2:** Comparison of patients with anti-Caspr1 IgG4 antibodies.

	Emilien Delmont (n = 2)	Andrea Cortese (n = 3)	Kathrin Doppler (n = 2)	Giuseppe Liberatore (n = 1)	Present report (n = 2)
**Age at onset (year)**	53 and 68	46 (24-57)	30 and 69	57	44 and 59
**Male sex**	2/2	2/3	1/2	0/1	1/2
**Subacute onset**	2/2	2/3	1/2	1/1	2/2
**Symptoms at onset**	NA	NA	Sensory deficits and severe pain, distal numbness	progressive proximal and distal motor involvement	Sensory deficits, severe pain with a cold sensation
**Triggering infection/vaccination**	0/0	1/3	2/2	NA	1/2
**Muscle weakness**	2/2	3/3	2/2	1/1	2/2
**more severe in distal**	0/0	3/3	1/2	NA	2/2
**Sensory deficiency**	2/2	3/3	2/2	1/1	2/2
**Sensory ataxia**	1/2	2/3	0/2	1/1	1/2
**Tremor**	0/2	1/3	0/2	1/1	0/2
**Pain**	1/2	0/5	2/2	0/1	2/2
**Neuropathic pain accompanying cold sense**	0/2	0/5	0/2	0/1	2/2
**Respiratory failure**	2/2	0/5	0/2	0/1	0/2
**Cranial nerve involvement**	2/2	1/3	0/2	NA	0/2
**CSF protein (g/L)**	12 and 2.5	4.26 (3.43–5.10)	5.2	NA	3.2 and 3.3
**Impairment**					
**ONLS (total)**	10 and 5	8 (8-9)	NA	NA	5 and 5
**MRC sum score**	NA	NA	58/60 ^*^	NA	56 and 56
**NCS features**					
**Prolonged DML**	NA	3/3	2/2	NA	2/2
**Reduced CV**	NA	3/3	2/2	NA	2/2
**Prolonged F wave**	NA	1/3	2/2	NA	2/2
**Conduction blocks**	NA	1/3	2/2	NA	0/2
**Temporal dispersion**	NA	1/3	1/2	NA	0/0
**Response to IVIG**	0/2	2/3	0/1	0/1	0/1
**Response to steroids**	0/1	2/3	1/1	0/1	2/2
**Response to PEX**	0/1	0/1	1/2	0/1	0/0
**Response to immune suppressors**	0/0	1/1	1/1	0/1	0/0

CSF, cerebrospinal fluid; CV, conduction velocity; DML, distal motor latency; IVIg, intravenous immunoglobulins; NCS, nerve conduction study; PEX, plasma exchange. *: The MRC sum score of patient 2 was not found in Doppler’s article.

NA, not applicable.

The modes of onset were subacute in both cases. Both patients had mild to severe muscle weakness in distal limbs and decreased sensory deficiency, including pinprick sensation and position sense which was similar to those reported previously in patients. One of the patients had a history of respiratory infection before the onset. They did not suffer respiratory failure, and none of them had cranial nerve involvement. Sensory ataxia appeared in both patient. Both patients experienced severe neuropathic pain as well as cold sensation and manifested coarse tremors in the hands. The NCS examination indicated that they both had prolonged DML, decreased CV, and CA, much like patients in other studies. Prolonged F waves and conduction blocks were also observed in both patients. We administered IVIG and high-dose glucocorticoid therapy to both patients. Whereas the IVIG did not work, the patients responded well to glucocorticoid therapy. However, it should be emphasized that shock therapy had slow action, taking about a month until clinical symptoms ameliorated distinctly. Notably, CSF exchange may rapidly reduce the CSF protein. This method could not maintain the reduced protein for a long time because CSF exchange just decreased the antibody concentration but not the antibody production so that it could only temporarily relieve the symptoms.

## Discussion

The Caspr1 clinical phenotype is distinguished by a subacute and severe onset, distal phenotype, sensory ataxia, and severe pain. The average age of onset for males was 44 (24-68) years old, and 61 (57-69) years old for females. Concerning the severe neuropathic pain, we stressed that the two China patients had severe pain accompanying cold sense, which may be a typical symptom for the anti-Caspr1 positive patient. And the two China patients manifested coarse tremors in the hands while Other studies recorded only one patient with the tremor. Respiratory failure and cranial nerve involvement are reported in Emilien’s two patients (4). All patients had high levels of protein according to the CSF tests. In nerve conduction tests, almost all patients had prolonged distal motor latency, decreased conduction velocity, and prolonged F wave latency. Three of the patients had conduction blocks, and three have temporal dispersion. In the lumbosacral plexus of one patient, the MRI scan showed increased signal intensity in the spinal roots and sciatic nerves on both sides. The enlargement of the spinal roots could be observed in the lumbosacral and brachial plexus in patient 2.

There were differences in clinical manifestation between the two China patients and those reported in other studies. Firstly, both patients in the current study had severe neuropathic pain accompanying cold sensation and coarse tremor in both hands, which had not been documented in previous studies. This disparity may be due to the differences in ethnic groups and living regions. Secondly, the isotype of antibodies in the past studies was IgG4, but we did not identify the isotype for the two patients in our study. Therefore, differences in isotypes of antibodies may be another reason for the observed disparities. Considering all the above, we suggest that pain with cold sense, coarse tremor in hands, and CSF protein levels greater than 3g/L may be the source of the distinct symptoms observed in patients with anti-Caspr1 autoantibodies in South China.

The treatment response of Caspr1 auto-antibodies differed significantly from that of seronegative CIDP. Previous studies found that two of three patients who received IVIG had a partial response (3); three of four patients who received steroids had a partial response (2, 3); one of the three patients who received plasma exchange had a good response (3), and two patients receiving immune suppressors had a good effect (2, 3). The IVIG was ineffective because its action mechanisms were blocking complement deposition and lessening inflammatory activity. However, the predominant subclass of autoantibodies is IgG4 which lacks access to complement C1q and thus cannot activate the inflammatory cascade (2). Consequently, CIDP patients with IgG4 autoantibody do not respond well to IVIg rendering rituximab as an option for these patients. Rituximab is a monoclonal antibody that specifically binds to the transmembrane antigen CD20 expressed on the surface of pre-B and mature B lymphocytes, triggering an immune response that mediates B cell lysis (2, 3, 9).

In the pathogenesis aspect, Andrea Cortese reported that mouse sciatic nerve segments were incubated *in vitro* with 10 μg of IgG4 or IgG1 fractions from Caspr1-positive patients for 3 hours, and IgG deposition was monitored. It was found that the IgG4 antibodies infiltrated the paranodal regions but igG1 did not (3). The level of antibody penetration across the paranodal region was comparable at one or three days after intraneural injection, and IgG4 deposition was detected only at the border of the nodes of Ranvier. In Kathrin Doppler’s study (2), the pain experienced by the patients could be explained by the binding of their IgG to TRPV1 immunoreactive dorsal root ganglia neurons. Kira (10) explained that the presence of Ig4 subclass antibodies in the bodies of patients, was due to their evolution in response to chronic antigenic stimulation, and block antibodies that alleviate allergic inflammation by interfering with the binding of allergen-specific IgE to allergens. Thus, environmental antigens that cross-react with nodal and paranodal proteins may warrant further investigation.

## Data Availability Statement

The original contributions presented in the study are included in the article/**Supplementary Material**. Further inquiries can be directed to the corresponding authors.

## Ethics Statement

The studies involving human participants were reviewed and approved by Ethics Committee of Nanfang Hospital, Southern Medical University. Written informed consent for participation was not required for this study in accordance with the national legislation and the institutional requirements.

## Author Contributions

CL and HJ: conception of the work, data acquisition, data interpretation, drafting, and revision of the manuscript for intellectual content. HZ, CY, and YL: data acquisition, data interpretation, and revision of the manuscript for intellectual content. YH: discussion and revision of the manuscript. All authors contributed to the article and approved the submitted version.

## Conflict of Interest

The authors declare that the research was conducted in the absence of any commercial or financial relationships that could be construed as a potential conflict of interest.

## Publisher’s Note

All claims expressed in this article are solely those of the authors and do not necessarily represent those of their affiliated organizations, or those of the publisher, the editors and the reviewers. Any product that may be evaluated in this article, or claim that may be made by its manufacturer, is not guaranteed or endorsed by the publisher.

## References

[1] Salzer JL Brophy PJ Peles E. Molecular Domains of Myelinated Axons in the Peripheral Nervous System. Glia (2008) 56(14):1532–40. doi: 10.1002/glia.20750 18803321

[2] Doppler K Appeltshauser L Villmann C Martin C Peles E Kramer HH. Auto-Antibodies to Contactin-Associated Protein 1 (Caspr) in Two Patients With Painful Inflammatory Neuropathy. Brain (2016) 139(Pt 10):2617–30. doi: 10.1093/brain/aww189 27474220

[3] Cortese A Lombardi R Briani C Callegari I Benedetti L Manganelli F. Antibodies to Neurofascin, Contactin-1, and Contactin-Associated Protein 1 in CIDP. Neurol Neuroimmunol Neuroinflamm (2019) 7(1):e639. doi: 10.1212/NXI.0000000000000639 31753915 PMC6935837

[4] Delmont E Brodovitch A Kouton L Allou T Beltran S Brisset M. Antibodies Against the Node of Ranvier: A Real-Life Evaluation of Incidence, Clinical Features and Response to Treatment Based on a Prospective Analysis of 1500 Sera. J Neurol (2020) 267(12):3664–72. doi: 10.1007/s00415-020-10041-z 32676765

[5] Koike H Kadoya M Kaida KI Ikeda S Kawagashira Y Iijima M. Paranodal Dissection in Chronic Inflammatory Demyelinating Polyneuropathy With Anti-Neurofascin-155 and Anti-Contactin-1 Antibodies. J Neurol Neurosurg Psychiatry (2017) 88(6):465–73. doi: 10.1136/jnnp-2016-314895 28073817

[6] Koike H Nishi R Ikeda S Kawagashira Y Iijima M Katsuno M. Ultrastructural Mechanisms of Macrophage-Induced Demyelination in CIDP. Neurology (2018) 91(23):1051–60. doi: 10.1212/WNL.0000000000006625 30429275

[7] Van den Bergh PYK van Doorn PA Hadden RDM Avau B Vankrunkelsven P Allen JA. European Academy of Neurology/Peripheral Nerve Society Guideline on Diagnosis and Treatment of Chronic Inflammatory Demyelinating Polyradiculoneuropathy: Report of a Joint Task Force-Second Revision. J Peripher Nerv Syst (2021) 26(3):242–68. doi: 10.1111/jns.12455 34085743

[8] Liberatore G De Lorenzo A Giannotta C Manganelli F Filosto M Cosentino G. Frequency and Clinical Correlates of Anti-Nerve Antibodies in a Large Population of CIDP Patients Included in the Italian Database. Neurol Sci (2022). doi: 10.1007/s10072-021-05811-0 35048233

[9] Tang L Huang Q Qin Z Tang X. Distinguish CIDP With Autoantibody From That Without Autoantibody: Pathogenesis, Histopathology, and Clinical Features. J Neurol (2021) 268(8):2757–68. doi: 10.1007/s00415-020-09823-2 32266541

[10] Kira JI Yamasaki R Ogata H. Anti-Neurofascin Autoantibody and Demyelination. Neurochem Int (2019) 130:104360. doi: 10.1016/j.neuint.2018.12.011 30582947

