# Supplementary material for: Two CIDP Variants Patients With Anti-Caspr1 Antibodies in South China
Source: Front Immunol. 2022 Mar 11;13:844036. doi: 10.3389/fimmu.2022.844036 (PMC8963365; doi:10.3389/fimmu.2022.844036)

Cell-based assay (CBA) assay: Human embryonic kidney cells were plated onto poly-L-lysine coated glass coverslips in 24-well plates at a density of 50 000 cells/wells and were transiently transfected with Caspr1 constructs (NM.003632.3) using pcDNA3.1-c-eGFP. The day after, cells were incubated for 24 h. Then cells were fixed, permeabilized, and incubated for 2 h with serum diluted at 1:10 in PBS at 37℃. After several washes with PBS, cells were incubated with goat antibodies against human IgG H&L (1:200;DyLight® 550) (ab96908) for 1 h at 37℃.

Patient 2

Patient 1


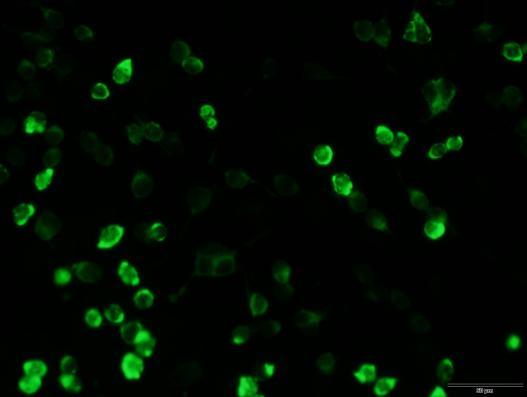

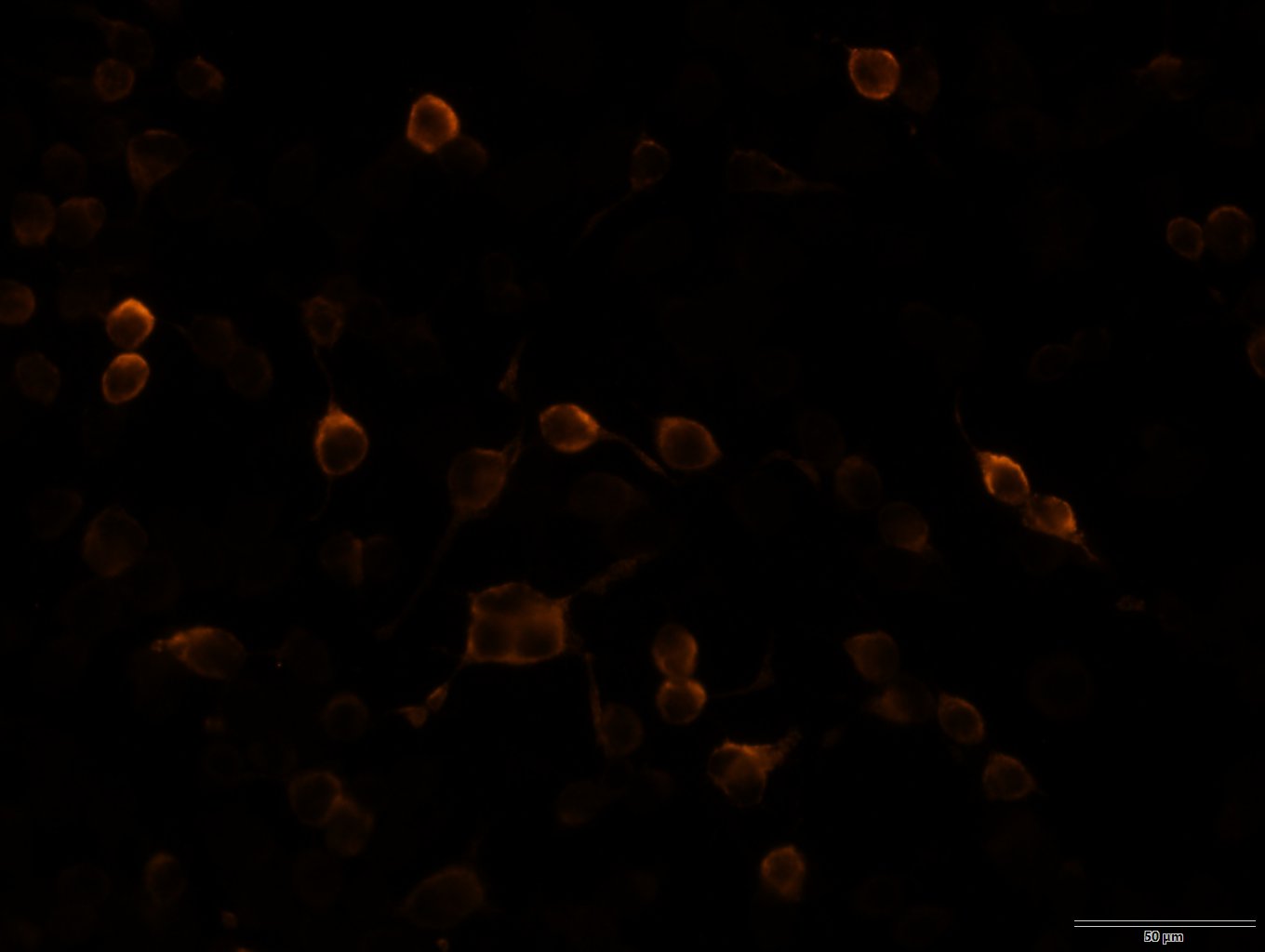

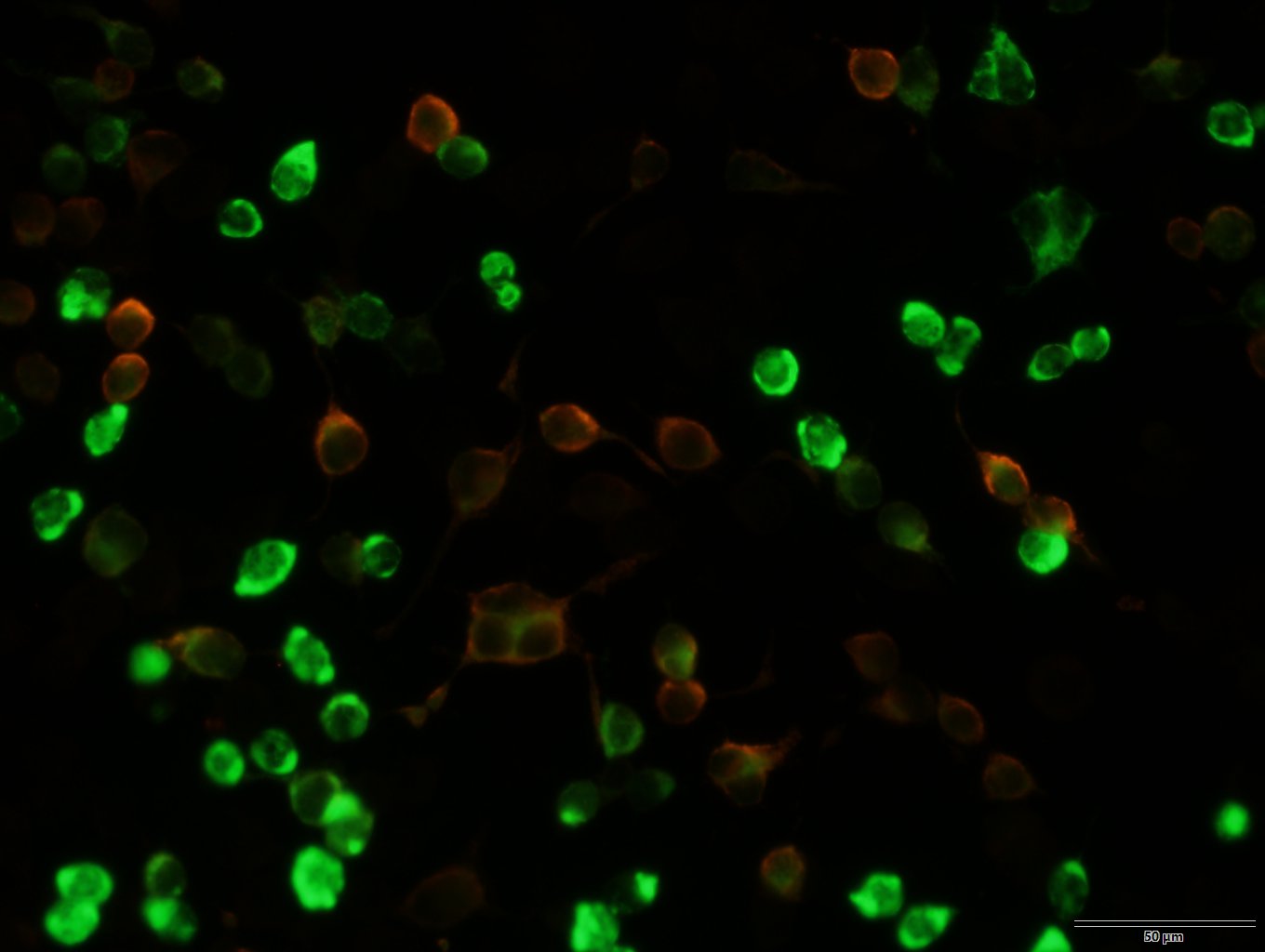

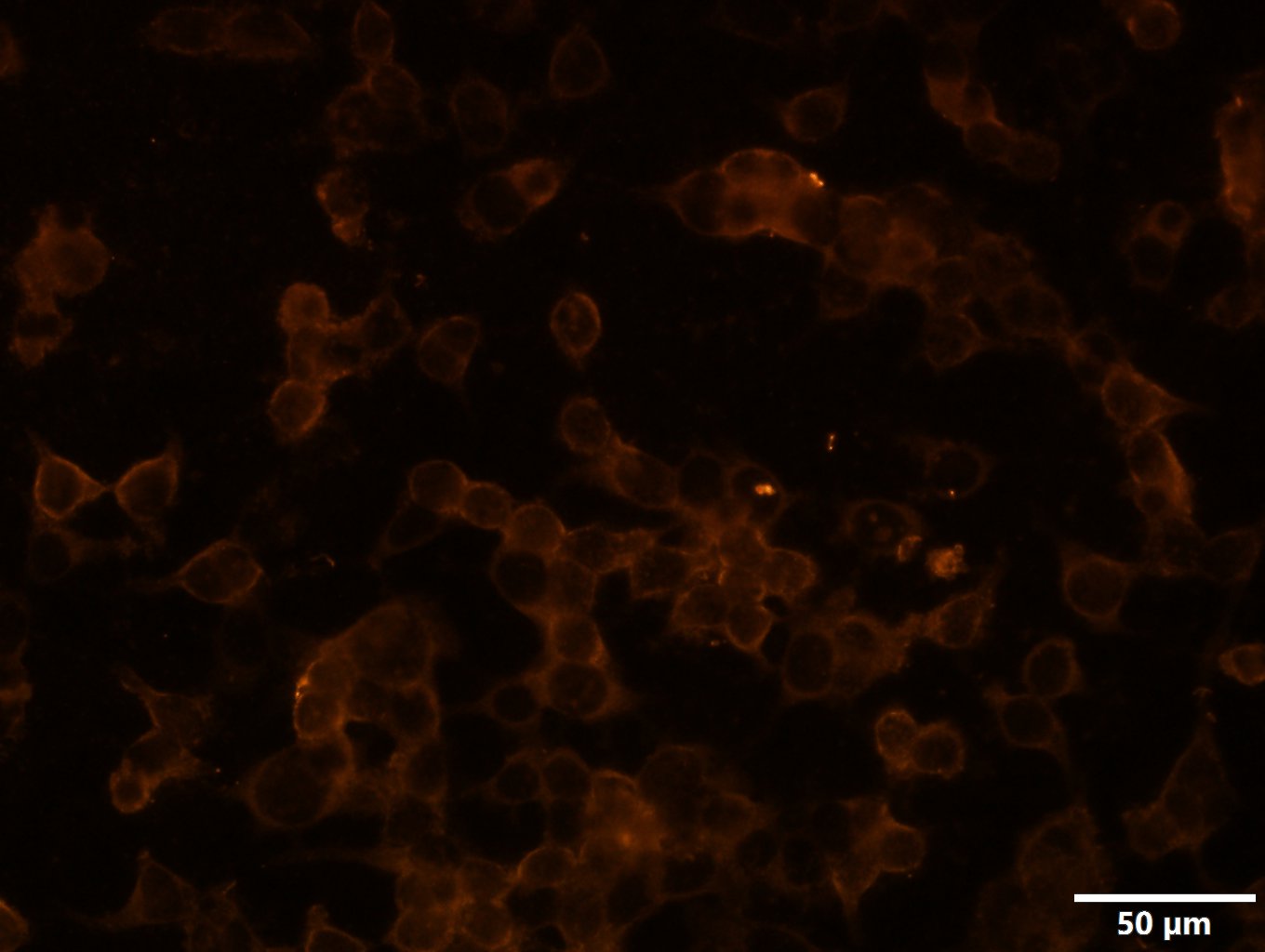

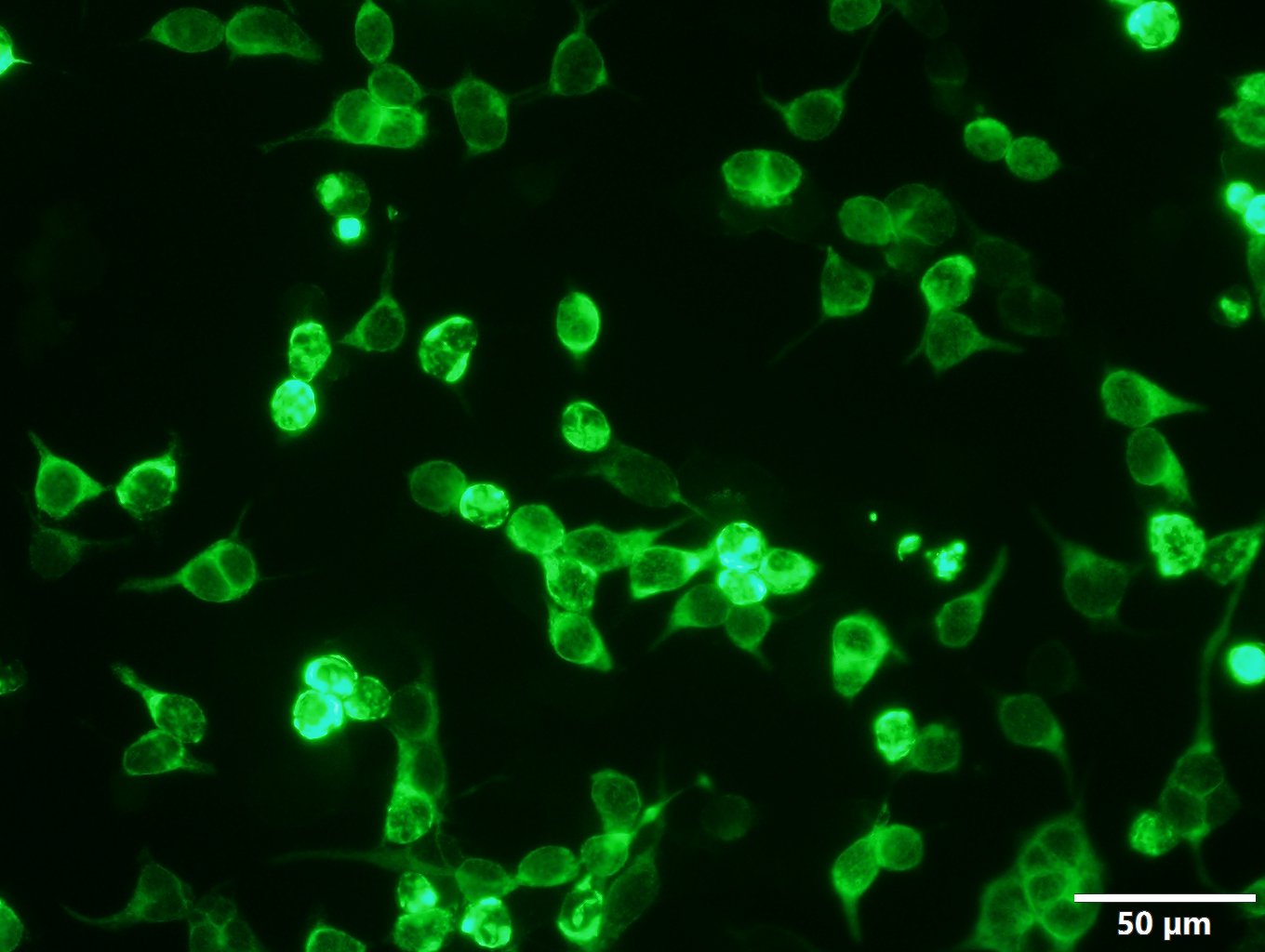

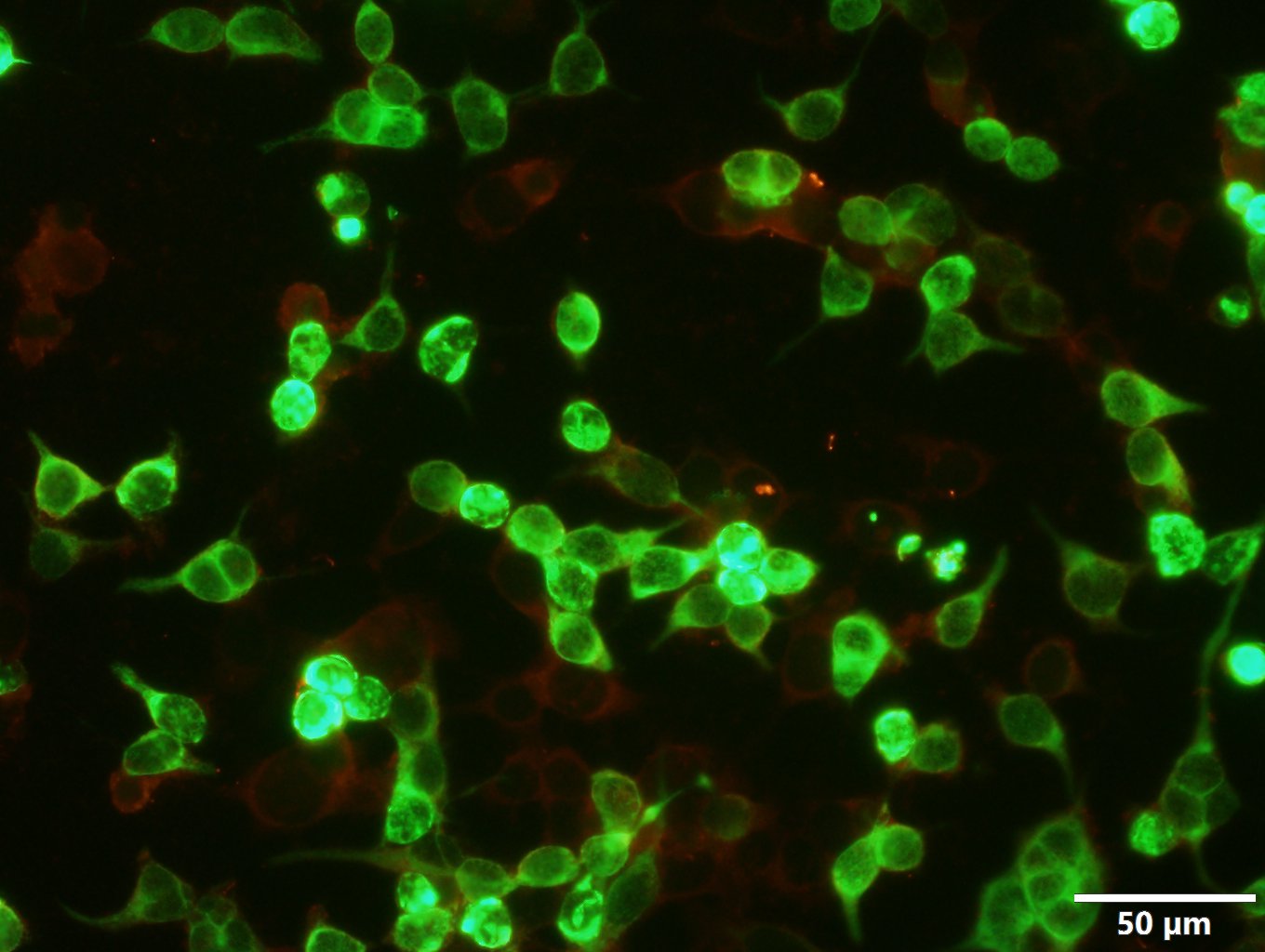

Supplement: Supplementary file 1 [file DataSheet_1.zip › CBA results.docx]
